# Left ventricular hemodynamic forces are altered in patients with dilated cardiomyopathy

**DOI:** 10.1186/1532-429X-17-S1-P282

**Published:** 2015-02-03

**Authors:** Jonatan Eriksson, Ann F Bolger, Tino Ebbers, Carl Johan Carlhall

**Affiliations:** Linköping University, Linköping, Sweden; Department of Medicine, University of California San Francisco, San Francisco, CA USA

## Background

Adverse cardiac remodeling is a key component of the failing heart. Increased diastolic wall stress plays a pivotal role in the development and progression of adverse cardiac remodeling. The forces generated by the left ventricular (LV) myocardium initiate blood flow, while the moving blood itself also exerts a force on the ventricular wall and heart valves. Abnormal hemodynamic forces may contribute to increased diastolic wall stress. We calculated LV hemodynamic forces from the moving blood, and hypothesized that these forces are mostly directed along the "mitral valve (MV) to apex axis" in the healthy LV, while the distribution is altered in myopathic LVs.

## Methods

**Figure 1:**
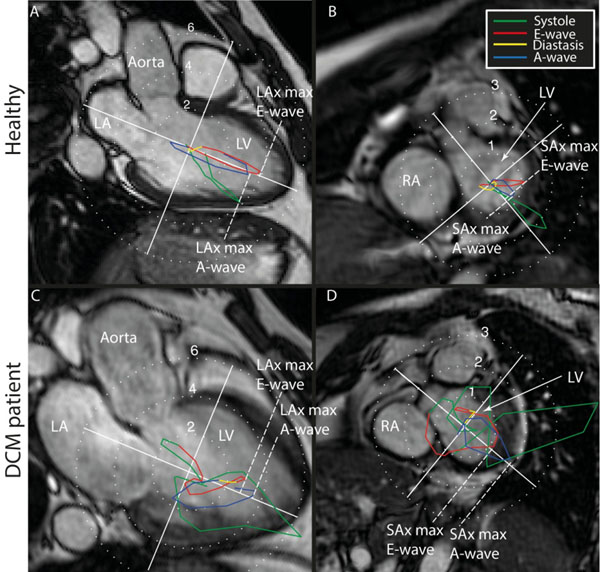
Hemodynamic forces [N] over the cardiac cycle, projected onto long axis (LAx) (A and C) and short axis (SAx) images (B and D). The cardiac cycle is colorcoded as: systole (green), early diastole (E-wave) (red), diastasis (yellow) and late diastole (A-wave) (blue). A-B) shows a healthy 54 y.o. male; C-D) shows a 43 y.o. male with dilated cardiomyopathy. The maximum values for E- and A-wave indicated by the dashed white lines in the LAx-direction (A and C) and in the SAx-direction (B and D) was used to calculate the "SAx-max/LAx-max force"-ratio. LA, Left Atrium; LV, Left Ventricle; RA, Right Atrium.

Ten dilated cardiomyopathy (DCM) patients (6 females) with mild to moderate ventricular dysfunction and ten healthy subjects (4 females) underwent a cardiac CMR examination at 1.5T (Philips Achieva), where morphological short- (SAx) and long-axis (LAx) images and 4D flow data was acquired. 4D flow MRI was acquired with retrospective cardiac gating; acquired temporal resolution 50.4ms and spatial resolution 3x3x3mm^3^. The LV was segmented from the morphological images using Segment (http://www.segment.heiberg.se). The LV pressure gradients were computed from the 4D flow data using the Navier-Stokes equations. By integrating the pressure gradients over the LV volume at every time frame, the LV hemodynamic force was calculated [Pedrizzetti, WCB 2014, Boston]. The ratio between the LV hemodynamic forces acting in the LV SAx and LAx directions was used to calculate the "SAx-max/LAx-max force-ratio" for the early (E-wave) and the late (A-wave) diastolic filling (figure 1).

## Results

There was no significant difference in age (DCM: 49±14 years vs Healthy: 48±15, P=0.98) or heart rate (61±11bpm vs 67±10, P=0.22) between the groups, while LV ejection fraction (41±5% vs 61±3, P=0.00), LV end-diastolic volume (177±33ml vs 137±15, P=0.003) and LV sphericity index (0.75±0.12 vs 0.56±6, P<0.0003) was significantly different. The "SAx-max/LAx-max"ratio (figure 1) was significantly larger at both E- (0.53±0.18 vs 0.19±0.1, p<0.0001) and A-wave (0.52±0.24 vs 0.32±0.11, P<0.03) in the DCM group compared to normals. This implies that MV to apex directed flow-based forces in normal LVs are redirected towards the SAx direction in DCM LVs.

## Conclusions

4D flow CMR data allow quantification of hemodynamic forces acting on the LV myocardium. The present data show that the distribution of hemodynamic forces is altered in LVs of DCM patients compared to LVs of healthy subjects.

## Funding

This study was funded by the Swedish Heart-Lung foundation, the Swedish Research Council and the European Research Council.

